# Wearable and Implantable Wireless Sensor Network Solutions for Healthcare Monitoring

**DOI:** 10.3390/s110605561

**Published:** 2011-05-26

**Authors:** Ashraf Darwish, Aboul Ella Hassanien

**Affiliations:** 1 Faculty of Science, Helwan University, Cairo, Egypt; 2 Faculty of Computers and Information, Cairo University, Cairo, Egypt; E-Mail: aboitcairo@gmail.com

**Keywords:** wireless sensor networks, body area networks, wearable sensors, implantable sensors, healthcare applications, biosensors, nanotechnology, privacy, security

## Abstract

Wireless sensor network (WSN) technologies are considered one of the key research areas in computer science and the healthcare application industries for improving the quality of life. The purpose of this paper is to provide a snapshot of current developments and future direction of research on wearable and implantable body area network systems for continuous monitoring of patients. This paper explains the important role of body sensor networks in medicine to minimize the need for caregivers and help the chronically ill and elderly people live an independent life, besides providing people with quality care. The paper provides several examples of state of the art technology together with the design considerations like unobtrusiveness, scalability, energy efficiency, security and also provides a comprehensive analysis of the various benefits and drawbacks of these systems. Although offering significant benefits, the field of wearable and implantable body sensor networks still faces major challenges and open research problems which are investigated and covered, along with some proposed solutions, in this paper.

## 1. Introduction

Wireless sensor network (WSN) technologies have the potential to change our lifestyle with different applications in fields such as healthcare, entertainment, travel, retail, industry, dependent care and emergency management, in addition to many other areas. The combination of wireless sensors and sensor networks with computing and artificial intelligence research have built a cross-disciplinary concept of ambient intelligence in order to overcome the challenges we face in everyday life [1]. One of the main challenges facing the World in recent years has been the increase in the elderly population in developed countries. According to the Population Reference Bureau [2], over the next 20 years, the 65-and over population in the developed countries will become almost 20% of the total population. Hence the need to provide quality care and service in these countries for a rapidly growing population of elderly people, while reducing the healthcare costs is an important issue for governments and health service providers in such countries. Wearable and implantable body sensor network systems are one tool to achieve this objective, as a prominent application in these areas is the integration of sensing and consumer electronics technologies which would allow people to be monitored during all their everyday activities.

Body sensor network systems can help people by providing healthcare services such as medical monitoring, memory enhancement, control of home appliances, medical data access, and communication in emergency situations [3,4]. Continuous monitoring with wearable and implantable body sensor networks will increase early detection of emergency conditions and diseases in at risk patients and also provide a wide range of healthcare services for people with various degrees of cognitive and physical disabilities. Not only the elderly and chronically ill, but also the families in which both parents have to work will benefit from these systems to provide high-quality care services for their babies and children.

**Figure 1.**
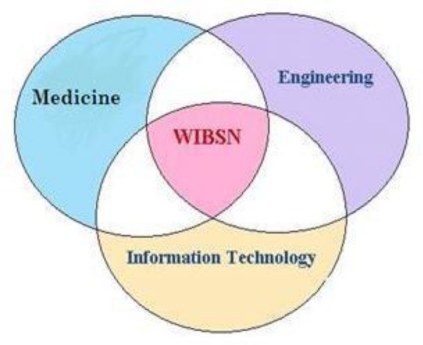
Interdisciplinary sciences for the future of WIBSN.

Researchers in various interdisciplinary fields such as computing, engineering, and medicine fields are working together in order to ensure that the broad vision of wearable and implantable body sensor networks (WIBSNs) for smart healthcare, as illustrated in Figure 1, can be fulfilled.

The importance of integrating large scale wireless telecommunication technologies such as 3G, Wi-Fi Mesh, and WiMAX, with telemedicine has already been addressed by some researchers. There are already several applications and prototypes for this purpose. For example, some of them are considered for continuous monitoring of people suffering from cognitive disorders like Alzheimer’s, Parkinson’s or similar diseases. Some papers focus on fall detection, posture detection and location tracking, while others make use of biological and environmental sensors to identify patients’ health status. There is also a significant research focus on the development of tiny wireless sensor devices, preferably integrated into fabrics or other wearable substances or implanted in the human body. The range of wearable and implantable biomedical devices will increase significantly in the next years, thanks to the improvements in micro-electro-mechanical systems (MEMS) technology, wireless communications, and digital electronics, achieved in recent years [5]. These advances have allowed the development of low-cost, low power, multi-functional sensor nodes that are small in size and can communicate over short distances, and tiny sensor nodes, which consist of sensing, data processing, and communicating components, and to take advantage of the idea of sensor networks based on collaborative effort of a large number of nodes.

The connections with leads and cables used in most current monitoring devices present obvious drawbacks in that they restrict the mobility of patients and, in addition, they may cause skin irritations or infections, and therefore contribute to deterioration of health conditions. Sensor networks thus represent a significant improvement compared to conventional sensors [6]. Despite the fact that a wireless connection is not a requirement for physiological parameter monitoring with implanted sensors, the issue mentioned above is one of the main motivations for this trend to use wireless technology in modern biomedical implanted systems [7]. Examples of physiological data collection platforms with wireless links include a wide range of biomedical applications. Coosemans *et al*. [8], for example, have described a system for continuous wireless intracavitary pressure monitoring of the bladder, while other authors have assembled neural prosthetic devices [9–11].

Tang *et al*. have described their vision [12] of a future when one device will be able to build a WSN with a large number of nodes, both inside and outside the human body that may be either predefined or random, according to the application. This vision can only be applied through the use of common communication protocols for wireless sensor networks. The standardized hardware and software architectures can support compatible devices, which are expected to significantly affect the next generation of healthcare systems. Some of these devices can then be incorporated into the wireless body area network (WBAN), providing new opportunities for technology to monitor health status [13].

This paper explores wearable and implantable body sensor network systems which provide rich contextual information and alerting mechanisms for abnormal conditions in continuous monitoring of patients at large. In addition, this paper evaluates the state of the art research activities and present issues that must be addressed to improve the quality of life through wearable and implantable body sensor networks. As well, we provide an overview of the recent trends on the future of intelligent applications for monitoring, not only from the wearable sensors perspective but also from the implantable sensor perspective. We also discuss the benefits that will be achieved and the outstanding research questions; in addition we introduce the open research problems that will be addressed with the development of sensor network based healthcare environment.

The remainder of this review is organized as follows: Section 2 briefly overviews the related work on wearable and implantable body sensor networks. Section 3 surveys wireless sensor networks as one of the promising technological cornerstones of ambient intelligence. Next, Section 4 presents the extension of wireless sensor networks to medical applications to turn them into wireless body sensor networks. Section 5 addresses the challenges and open research problems for wireless body area networks and healthcare applications. Finally Section 6 draws the conclusions and future work.

## 2. Related Work

Many implementations of body area networks used for body sensor communication have recently been reported in the literature [14–16]. A number of these researches focus mainly on on-body MAC layer issues. The system in [13] uses a slotted multipoint-to-point architecture in which the data from multiple on-body sensors are sent to a sink node in a collision free manner. The transmission slots are synchronized using beacon signals periodically sent out by a pre-designated sink node. The mechanisms reported in [14] involve the use of an on-body adaptation of the standard IEEE 802.15.4/ZigBee based MAC. The work reported in [15,16] investigated on-body MAC-routing cross-layer issues via distributed transmission coordination in the presence of specific routing structures. In [15], the authors presented an energy-efficient slotted MAC in the presence of a Wireless Autonomous Spanning tree Protocol (WASP) that is used for on-body packet routing.

The protocol in [16] adopts a similar tree-based cross-layer approach, but is designed specifically for reducing packet delivery delays over an on-body spanning tree. This protocol also handles body mobility by adaptively re-constructing and maintaining the spanning tree used for packet routing.

From the on-body routing standpoint, most of the existing WBANs systems adopt star or tree topologies on a connected graph; meaning a physically connected end-to-end path between any pair of on-body sensors is assumed at any given point in time. However, these models do not apply for the targeted DTN routing paradigm in this paper, which handles topology partitioning leading to scenarios in which end-to-end physical connectivity between node pairs may not be present at times. Such partitioning is mainly due to the ultra short range RF transceivers used.

The knowledge based strategies are typically for single copy forwarding and they make use of information about connectivity dynamics to make efficient forwarding decisions. The hybrid approaches [14,16,17] combine replication and knowledge based strategies. The general principle behind these approaches is as follows: when a node with a packet to be forwarded encounters another node, the forwarding rule should determine if the packet (or a copy of the packet) should be transferred to that node or it should continue to be buffered. The rule is based on the estimate whether the encountered node is more likely than the forwarding node to visit the destination.

The above mechanisms are all applied to networks spanning across local to wide areas, with a few extending all the way up to the inter-planetary scale. The objective of the work described in [18] is to apply the key DTN routing concepts, as identified above, in an ultra short-range body area environment. The challenge is to develop mechanisms for capturing the locality of on-body node movements caused by human postural mobility.

WBANs provide efficient communication solutions to ubiquitous healthcare systems. Health monitoring, telemedicine, military, interactive entertainment, and portable audio/video systems are some of the applications where WBANs can be used. Khan *et al*. [19] have presented a comprehensive work on the applications of WBANs in smart healthcare applications, including epileptic seizure warning, glucose monitoring, and cancer detection. In this paper, the authors highlighted a number of projects that enable WBANs to provide unobtrusive long-term healthcare monitoring with real-time updates to a health center. Mark *et al*. [20] presented BASNs as systems enabling human-centric sensing for a variety of intriguing applications in healthcare, fitness, and entertainment, but such networks must demonstrate enough value for users to overcome inhibitions related to inconvenience, invasiveness, and general discomfort. Authors described wearable technologies that will “silently monitor” heart rhythm, detect irregularities, and alert emergency personnel in the event of a heart attack. This vision, not far removed from current research efforts, illustrates the promise of WBANs in this important area.

## 3. Wireless Sensor Networks

### 3.1. Background and Overview

Embedded electronic systems are widespread or pervasive; from alarm clocks to PDAs, from mobile phones to cars, almost all the devices we use on a daily basis are controlled by embedded or internal electronics. In relation with the recent advances in technology more than 99% of the microprocessors produced today are used in such embedded systems, and lately the number of embedded systems in use has developed and increased. The concept of ambient intelligence reflects the vision that technology will not only develop but be embedded, invisible, and fully hidden in our natural surroundings, but present whenever we need it, enabled by simple and easy interactions [21,22]. Ambient intelligence has been defined by the Advisory Group to the EU Information Society Technology Program (ISTAG) as “the convergence of three major key technologies: ubiquitous computing, ubiquitous communication, and interfaces adapting to the user”. In the near future ubiquitous computing and communication will attract more interest and research.

Wireless sensor networks [23,24] are usually considered one of the technological foundations of ambient intelligence. Agile, low-cost, ultra-low power networks of sensors can collect a huge amount of important information from the surrounding environment. Using a biological analogy, sensor networks can be viewed as the sensory system of the intelligent environment of the human body. Sensor networks are irregular clusters of communicating sensor nodes, which collect and process information from onboard sensors, and they can share some of this information with neighboring or surrounding nodes or even with nearby data collection stations.

Current sensor network applications in ambient intelligence range from the environmental monitoring of ecosystems and industrial processes for tracking assets and people to the maintenance of buildings, *etc*. [25]. In addition, each application domain has different requirements and restrictions based on the nature of the problem. In this review, we will focus on one specific driver application, namely monitoring of humans based on wearable and human body implantable sensors to expand the role of such technologies in healthcare and to overcome new challenges from Nature on human health. There are some important reasons for this choice. First, human monitoring is an extremely important field and important for all of us. Second, the scope of human monitoring application poses new challenges such as requisites for unobtrusiveness, security and low energy consumption, *etc*. Finally, this is one of the ambient intelligence application fields where research and development are the most active areas.

The design, implementation, and deployment of WSNs requires input from a wide range of disciplines such as medicine, engineering, and computing, as mentioned earlier in the paper, in addition to consideration of the numerous application-specific constraints. In the last five years, significant progress has been made in the development of WSNs, and some WSN-based commercial products have already appeared on the market. We refer readers interested in studies on software and network architectures to any of the many excellent surveys that have recently been published on these topics [26,27].

A sensor network consists of a large number of sensor nodes, which are deployed either inside the phenomenon to be monitored or very close to it. Sensor networks represent a significant improvement over traditional sensor networks, which are deployed in the following two ways [28]:

- Sensors can be located far from the actual phenomenon to be monitored. With this approach, large sensors that use some complex methods to distinguish the targets from environmental noise are required.
- Several sensors that perform only sensing can be deployed. The positions of the sensors and communications network topology are carefully designed. They transmit time series of data about the phenomenon to central nodes where computations are performed and data are fused.

There is another unique feature of sensor networks that is the cooperative effort of sensor nodes. Sensor nodes are equipped with an on-board processor. Instead of sending raw data to other nodes responsible for the fusion, sensor nodes use their processing abilities to locally carry out simple computations and transmit only the required and partially processed data.

### 3.2. Wireless Sensor and *Ad Hoc* Networks

Wireless *ad hoc* networking techniques can be deployed for wireless sensor network applications. Although many protocols and algorithms have been proposed for traditional wireless *ad hoc* networks, they are not well suited for the unique features and application requirements of sensor networks. In this subsection we briefly compare sensor and *ad hoc* networks to emphasize the important role of wireless sensor networks, especially in monitoring of the human body. To illustrate this point, the differences between sensor networks and *ad hoc* networks can be summarized as follows:

- Numbers of Sensor Nodes: the number of sensor nodes in a sensor network can be many orders of magnitude higher than the nodes in an *ad hoc* network.
- Efficiency: sensor nodes are densely deployed.
- Failure: sensor nodes are prone to failures.
- Network Topology: the topology of a sensor network changes very frequently.
- Communication Ability: sensor nodes mainly use broadcast communication paradigm whereas most *ad hoc* networks are based on point-to-point communications.
- Computing: sensor nodes are limited in power, computational capacities, and memory.
- Cost: Sensor nodes may not have global identification because of the large amount of overhead and large number of sensors.

### 3.3. WSN Applications

Recent advance in the integration and miniaturization of physical sensors, embedded microcontrollers and radio interfaces on a single chip; wireless networking; and micro-fabrication have provided a new generation of wireless sensor networks suitable for many applications. The features described in the previous section provide a wide range of applications for sensor networks. Wireless sensor networks can be used for military applications, habitat monitoring, machine health monitoring and guidance, traffic pattern monitoring and navigation, plant monitoring in agriculture [29], and infrastructure monitoring. One of the most exciting applications is health monitoring [30,31]. In this paper we focus on healthcare applications, and mainly on wearable and implantable body sensor networks. A number of physiological sensors that monitor vital signs, environmental sensors, and a location sensor can be integrated into a wearable wireless body area network (WWBAN) [32]. In addition, the concept of micro-sensing and wireless connection of network nodes promises many new application areas. We have categorized the applications into military, environment, health, home and other commercial areas. It is possible to extend this classification with more categories such as space exploration, chemical processing, disaster relief, and so on. We briefly describe these applications and for more details, the reader should refer to [33].

#### 3.3.1. Military Applications

Wireless sensor networks can be a part of military command, control, communications, computing, intelligence, surveillance, reconnaissance and targeting systems. The rapid deployment, self-organization and fault tolerance characteristics of sensor networks make them a very promising sensing technique for military applications. Some of the military applications of sensor networks are monitoring friendly forces, equipment and ammunition; battlefield surveillance; reconnaissance of opposing forces and terrain; targeting; battle damage assessment; and nuclear, biological and chemical attack detection (recently is considered as one of the critical types of attacks) and reconnaissance.

#### 3.3.2. Environmental Applications

Another important area of wireless sensor networks are environmental applications which include smart homes, tracking the movements of birds, small animals and insects; monitoring environmental conditions that affect crops and livestock; irrigation; macroinstruments for large-scale earth monitoring and planetary exploration; chemical/biological detection; precision agriculture; biological, earth, and environmental monitoring in marine, soil, and atmospheric contexts; forest fire detection; meteorological or geophysical research; flood detection; bio-complexity mapping of the environment; and pollution study.

#### 3.3.3. Home Applications

Home automation: as technology advances, smart sensor nodes and actuators can be incorporated into appliances, such as vacuum cleaners, micro-wave ovens, and refrigerators. These sensor nodes inside the devices can communicate with each other and with the external network via the Internet or satellite. They allow end users to control home devices locally and remotely more easily and can be used as alarms for disasters at homes.

#### 3.3.4. Commercial Applications

Some of the commercial applications are monitoring material fatigue; building virtual keyboards; managing inventory; monitoring product quality; constructing smart office spaces; environmental control in office buildings; robot control and guidance in automatic manufacturing environments such as interactive toys; interactive museums; factory process control and automation; monitoring disaster area; smart structures with sensor nodes embedded inside; machine diagnosis; transportation; factory instrumentation; local control of actuators; detecting and monitoring car thefts; vehicle tracking and detection; as well as instrumentation of semiconductor processing chambers, rotating machinery, and wind tunnels.

#### 3.3.5. Healthcare Applications

Some of the health applications of sensor networks involve providing interfaces for the disabled, integrated patient monitoring, diagnostics, drug administration in hospitals, telemonitoring of human physiological data, and tracking and monitoring doctors and/or patients inside a hospital [34–36]. We will briefly explore some capabilities of WSN for healthcare monitoring [37] as we focus in this paper on wearable and implantable body area networks.

- **Telemonitoring of Human Physiological Data**: The physiological data collected by sensor networks may be stored for a long period of time, and can be used for medical investigations when needed. In addition, the installed sensors can also monitor and detect the behavior of elderly people. As an example, a health smart home was designed by the Faculty of Medicine in Grenoble-France to check the feasibility of such systems [38].
- **Tracking and Monitoring Doctors and Patients inside a Hospital**: Each patient has a small sensor node attached to them. Sensors vary based on their functions and each sensor node has its own specific task to perform. For example, one sensor node may be detecting the heart rate while another is detecting the blood pressure. Doctors can also carry a sensor node, which allows other doctors to locate them within the hospital.
- **Drug Administration in Hospitals**: If sensor nodes can be attached to medication, the chance of getting and prescribing the wrong medication to patients can be minimized. Thus, patients will have sensor nodes that identify their allergies and required medication. Computerized systems as described in [39] have shown that they can help minimize the side effect of drugs.

## 4. Wireless Body Area Networks (WBANs)

The expansion of WSNs for medical applications is increasingly turning these technologies into body sensor networks (BSNs). The biosensors can record electrocardiograms, electromyographs, measure body temperature and blood pressure, electro-dermal activity, among other healthcare parameters. For example, accelerometers can be used to sense heartbeat rate, movement or even muscular activity.

Thanks to portable devices such as cellular phones and MP3 players that have became popular; people have started to routinely carry these devices around their body. In 2001, Zimmerman [40] studied how such electronic devices operate at or near the human body. He used the term wireless personal area network (PAN) and used it as a communication channel for intra-body communications. Later around 2001, the term PAN has been changed to body area network (BAN) to include the applications and communications using wearable (on/around) and implantable (in) [40]. A sensor network that senses health parameters thus becomes a body sensor network (BSN). A wireless body-area network (WBAN) is a special purpose wireless-sensor network that incorporates different networks and wireless devices to enable remote monitoring in various environments.

One of the targeted applications of WBANs is in medical environments where conditions of a large number of patients are constantly being monitored in a real-time environment. Wireless monitoring of physiological signals of a large number of patients is one of the current needs in order to deploy a complete WSN in healthcare systems. The main goal of WBANs is to provide biofeedback data, the ability to continuously monitor health parameters such as body/intra-body temperature, heartbeat rate, arterial blood pressure, in an unobtrusive and efficient way [41].

Human health monitoring [7–10] is emerging as a prominent application of the embedded sensor networks. A number of tiny wireless sensors are strategically placed on/in a patient’s body, to create a WBAN [42]. A WBAN can monitor vital signs, providing real-time feedback to allow many patient diagnostics procedures using continuous monitoring of chronic conditions, or progress of recovery from an illness. Recent technological advances in wireless networking promise a new generation of wireless sensor networks suitable for those on-body/in-body networked systems.

**Figure 2.**
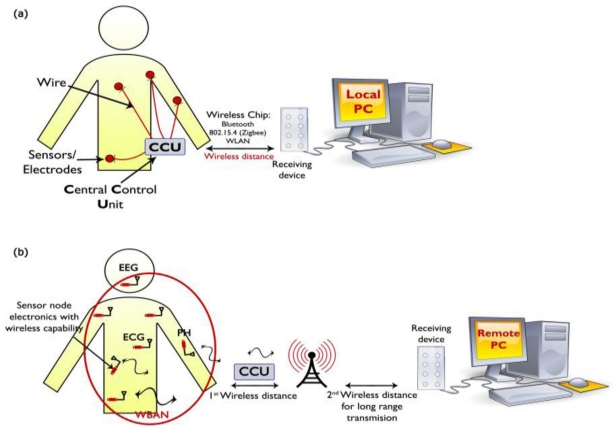
A typical WSN system for detecting and transmitting signals from a human body: **(a)** current application of healthcare sensor network and **(b)** future application of healthcare sensor network targeted by wireless body area network.

Data acquisition across such sensors can be point-to-point or multipoint-to-point, depending on specific applications. While the distributed detection of an athlete’s posture [43] would need point-to-point data sharing across various on-body sensors, applications such as monitoring vital signs as shown in Figure 2, will require all body mounted and/or implanted sensors [44,45] to route data multipoint-to-point to a sink node, which in turn can relay the information wirelessly to an out-of-body server. Data transaction may be also real-time or non-real-time. Although the patient monitoring type of applications requires real-time packet routing, monitoring an athlete’s physiological data can be collected offline for processing and analysis purposes.

A typical wireless body area network is composed of a number of miniature, lightweight, low-power sensing devices, management electronics and wireless transceivers. As an indispensible part of the system, the power supply for these components should be small-sized, lightweight, environmentally-friendly and long lasting as well.

**Figure 3.**
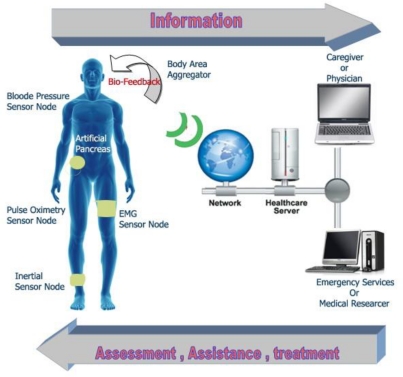
WBAN and its environment.

Unlike conventional WSNs, WBANs consists of smaller, less nodes and less space covered and fewer opportunities for redundancy, as shown in Figure 3. Scalability can lead to inefficiencies when working with the two to ten nodes typical of a WBAN. Adding sensor and path redundancy for solving node failure and network congestion problems cannot be a viable mechanism for a BASN seeking to minimize form factor and resource usage. WBANs also have a distinctly hierarchical nature. They capture large amount of data constantly and naturalistically that microprocessors should process to extract the needed information. Data processing must be hierarchical to exploit the asymmetry of resources, to maintain system efficiency, and ensure the availability of data, if necessary [20].

The application of WBANs in a medical area consists of wearable and implantable sensor nodes that can sense biological information from the human body and transmit it over a short distance wirelessly to a control device worn on the body or placed in an accessible location. The sensor electronics must be miniaturized, low-power and detect medical signals such as electrocardiograms, photoplethysmograms, electroencephalography, pulse rate, pressure, and temperature. The gathered data from the control devices are then transmitted to remote destinations in a wireless body-area network for diagnostic and therapeutic purposes by including other wireless network for long-range transmission.

Currently the monitoring devices that are used in medical centers are not completely wearable because their electronics are bulky and wires are used for connections to multiple sensors. As shown in Figure 1(a), the current application of a sensor network that used in some modern medical centers. A wireless control unit is used to collect information from sensors through wires and transmits it to a remote station for monitoring. The control unit is cumbersome and using wires is not recommended for the comfort of patients. As shown in Figure 1(b), the future medical sensor network requires miniaturized and wearable sensor nodes that can communicate with the receiving device wirelessly.

### 4.1. Requirements for Wireless Medical Sensors in WBANs

Wireless medical sensors should satisfy the following main requirements such as wearability, reliability, security, and interoperability [46]:

#### 4.1.1. Wearability

To achieve non-invasive and unobtrusive continuous monitoring of health, wireless medical sensors must be lightweight and small. Size and weight of sensors are mainly determined by the size and weight of batteries [47,48]. But, a battery’s capacity is directly proportional to its size. We can expect that further development of technology and advances in miniaturization of integrated circuits and batteries will help developers to improve medical sensor wearability and the user’s level of comfort.

#### 4.1.2. Reliable Communication

Reliable communication in WBANs is of paramount importance for medical applications that rely on WBANs. The communication needs of different medical sensors depending on the need of sampling rates, from less than 1 to 1,000 Hz. One approach to improve reliability is to move beyond telemetry by performing processing of the sensor signal. For example, instead of sending raw electrocardiogram data from sensors, we can perform feature extraction on the sensor, and transfer only information about an event. In addition to reducing the high demands on the communication channel, the reduced communication requirements saves on total energy expenditures, and consequently increases battery life. A careful trade-off between communication and computation is crucial for optimal system design.

#### 4.1.3. Security

Another important issue is the security of the entire system of WBANs. The problem of security occurs on all three levels of a WBAN-based telemedicine system. At the lowest level, wireless medical sensors must meet the requirements of privacy provided by the law for all medical devices and should ensure data integrity. Although the key establishment, authentication and data integrity are difficult tasks in limited resources of medical sensors, a relatively small number of nodes in a typical WBAN and communication ranges make these tasks achievable.

#### 4.1.4. Interoperability

Wireless medical sensors should allow users to easily build a robust WWBAN depending on the user’s state of health. Standards governing that interaction of wireless medical sensors will help vendor competition and eventually lead to more accessible systems.

### 4.2. Wearable Wireless Body Area Network (WWBAN)

WSN technology has the potential to offer a wide range of benefits to patients, medical staff, and society through continuous monitoring in an ambulatory setting, early detection of abnormal conditions, supervised rehabilitation, and potential discovery of knowledge through data mining of all gathered information. This subsection shows how to use the architecture and mechanism of WWBANs as one of the main points of this paper, as well as key infrastructure enabling unobtrusive, continuous, daily monitoring of health. In addition, this subsection describes some important implementation issues. Finally, we give some applications of wearable sensors as in smart shirt (smart life) and biopotential acquisition system application.

Wearable health monitoring systems allow the individual to follow closely the changes in her or his vital functions and provide feedback for maintaining optimal health status. If integrated into the telemedicine system, such systems can alert medical personnel when life-threatening changes occur. In addition, patients may benefit from continuous long-term monitoring as a part of a diagnostic procedure. We can achieve optimal maintenance of a chronic condition, or can be monitored in the recovery period after the acute event or surgical procedure. Long-term health monitoring can capture the diurnal and circadian variations in physiological signals. These changes, for example, are a very good indicator of cardiac recovery of patients after myocardial infarction [49]. Long-term monitoring can also confirm adherence to treatment guidelines or help monitor the effects of drug therapy. Other patients may also benefit from these systems; for example, monitors can be used during physical rehabilitation after hip or knee surgeries, stroke rehabilitation, or brain trauma rehabilitation.

The use of wearable sensors for monitoring various health-related biometric parameters in everyday activities is attracting more interest recently. Many people are familiar with the use of devices such as wearable heart rate monitors and pedometers for medical reasons or as part of a fitness regime. Interest in the use of such wearable systems for personal health and rehabilitation has increased as part of a wider initiative for increasing the input of the individual or patient in their own care [50]. It is believed that this could help in reducing the strain put on healthcare systems of aging populations, rising costs and increasing incidence of chronic diseases requiring long term care.

To date the focus has been on the use of wearable sensors to convert physical biometrics such as heart or respiratory rate into electrical signals. For instance, EU funded projects such as WEALTHY and My Heart use sensorised cotton shirts to measure respiratory activity, electrocardiograms, electromyograms and body posture [51–53]. In addition, NASA [54] is developing a wearable patch to control heart rate, blood pressure and other physiological parameters for astronauts [52]. Other systems include the Lifeshirt, developed by Vivo metrics, the body monitoring system developed by Body Media and the Nike-Apple iPod Sports kit [55].

While the growing success of sensors that monitor the physical properties, relatively little has been done in the field of wearable chemical sensors that can be used for real-time daily monitoring of bodily fluids such as tears, sweat [56], urine and blood. Although some achievements, such as the development of the Glucowatch and other wearable systems for monitoring glucose levels in diabetics, the widespread use of chemical sensors has been complicated by several factors that are difficult to overcome. These include sample collection and delivery, sensor calibration, wearability and security issues [32,55].

**Figure 4.**
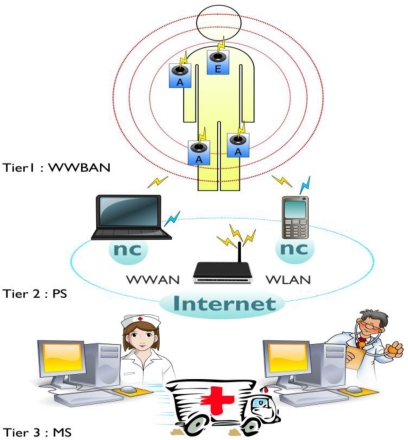
Typical WWBAN architecture.

WWBANs are a key part of a multi-stages or multi-layered system of telemedicine as shown in Figure 4. The architecture includes the first stage to cover the number of wireless nodes of medical sensor that are integrated into WWBANs. Each sensor node sense, sample, and process one or more physiological signals. For example, an electrocardiogram sensor can be used to monitor heart activity, an electromyogram sensor for monitoring muscle activity, an electroencephalogram sensor for monitoring brain electrical activity, a blood pressure sensor for monitoring blood pressure, a tilt sensor for monitoring trunk position, and a breathing sensor for monitoring respiration; and motion sensors can be used to discriminate the user’s status and to estimate her or his level of activity.

The second stage includes the personal server application that runs on a personal digital assistant, cell phone or home personal computer. The personal server is responsible for a number of targets, providing an interface for wireless medical sensors, users and the medical server. The interface to the WWBAN includes network configuration and management features. The network configuration includes the objectives: a sensor node registration (type and number of sensors), initialization (e.g., to specify sampling frequency and mode of operation), customization (e.g., to run user-specific calibration or user-specific signal processing procedure upload), and security settings communication.

Once the WWBAN network is configured, the personal server application manages the network, taking care of channel sharing, time synchronization, retrieve and process data, and fusion of the data. Based on synergy of information from different medical sensors the personal server application must determine the u status of the user and his health condition and to provide feedback through a user-friendly and intuitive graphical or audio user interface. Finally, if a channel of communication for the medical server is available, the personal server establishes a secure connection to the medical server and sends reports that can be integrated into the user’s medical record. However, if a connection between the personal server and the medical server is unavailable, the personal server must be able to store data locally and initiate data uploads when the connection becomes available.

The third stage includes a medical serve access via the Internet. In addition to the medical server, the last tier may include other servers, such as informal caregivers, commercial providers of healthcare and even emergency servers. The medical server typically runs a service that sets up a communication channel for the personal server to users, collects reports from user, and integrates the data in the medical record of the user. Service can make recommendations, and even the question of warning, if the reports seem to indicate an abnormal state.

#### 4.2.1. Wearable Smart Shirt Application

**Figure 5.**
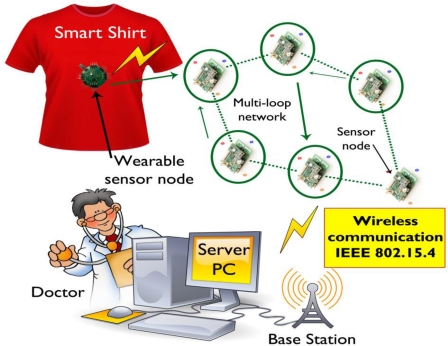
The system architecture with a wearable smart shirt.

A number of wearable physiological monitoring systems have been developed to monitor the health status of the individual wearer or the elderly [17,57–61]. A wearable physiological monitoring system called ‘Smart Vest’ to monitor various physiological parameters such as electrocardiograms, photoplethysmographs, heart rate, blood pressure, body temperature and galvanic skin response has been developed [58]. Figure 5 shows the overall system architecture of the wearable smart shirt for ubiquitous health and activity monitoring, which consists of a shirt with integrated wireless sensor nodes, a base station and a server personal computer for remote monitoring.

The smart shirt can measure electrocardiogram and acceleration signals for continuous monitoring and real time healthcare was designed and developed in [59]. The shirt contains sensors for continuous monitoring health data and conducting fabrics acting as electrodes to capture the body signals. The measured physiological electrocardiogram data and physical activity data are transferred to an *ad-hoc* network using the IEEE 802.15.4 communication standard for base-stations and a server PC for remote monitoring. A wrist worn wearable medical monitoring and alert system targeting high-risk cardiac/respiratory patients has been developed to monitor physiological parameters such as electrocardiograms, heart rate, blood pressure, skin temperature, *etc*. [17].

#### 4.2.2. Wearable Biopotential Acquisition System Application

Experts point out that just in the U.S more than 98,000 people die each year in hospitals due to the medical errors; such as diagnostics and treatment errors, which are normally associated with the conditions and disadvantages of systems that make people commit mistakes. Furthermore, the cost of healthcare delivery has been steadily raising. An important and expensive part of the existing healthcare systems is the monitoring of the biopotential signals, such as electroencephalograms, electrocardiograms, and electromyograms. During the monitoring of the biopotential signals, patients are connected to bulky and mains-powered devices, which not only reduces their comfort and mobility, but also increases the cost due to the fact that the monitoring must always take place in a hospital setting under the supervision of medical personnel.

On the other hand, there is tremendous interest in the use of biopotential signals in non-clinical applications. Some examples of these non-clinical applications in the case of electroencephalogram monitoring may be the human-computer interfaces and games, where the computer functions can be controlled through the user’s mind or the characters in the game can respond to the thoughts of the player. In addition, electromyogram signals can be monitored to improve the performance of athletes and electrocardiogram signals can be used for ease of monitoring. However, there are different challenges for clinical and nonclinical applications in terms of the biopotential acquisition systems. Although the main problem for the clinical applications is the quality of the signal, which is acquired with the acquisition system, the non-clinical applications also require that the biopotential monitoring device should be comfortable, miniaturized, battery powered, and unobtrusive. Furthermore, it should also have long-term power autonomy and wireless communication, so that a true ambulatory biopotential monitoring system can be achieved.

**Figure 6.**
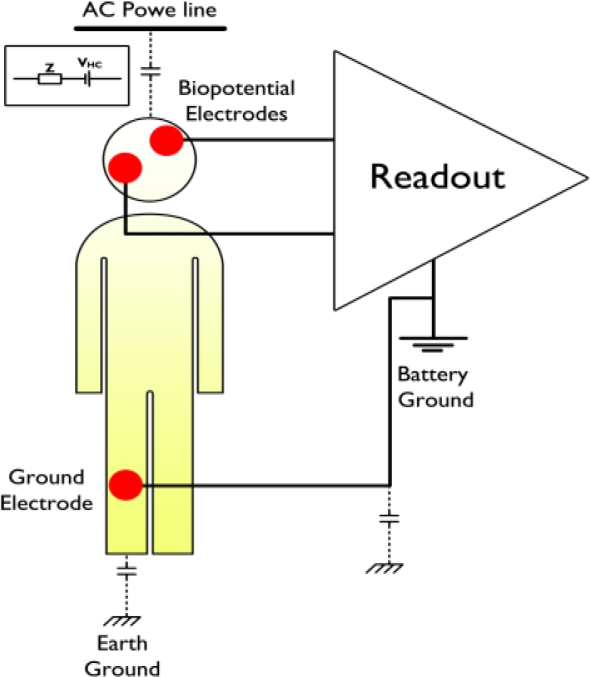
A biopotential readout circuit linked to the biopotential electrodes to extract the electroencephalogram signals.

The extraction of biopotential signals can be achieved by linking electrodes to the human body. These electrodes are in fact act as transducers and convert the ionic current in the human body into electronic current so that the readout circuit can amplify the biopotential signals [62,63]. Figure 6 shows a typical configuration that uses a readout circuit to extract biopotential signals from the human body using biopotential electrodes to which the inputs of the readout circuit are connected.

### 4.3. Implantable Wireless Body Area Network (IWBAN)

To measure heath parameters, biosensors must be in close contact with the skin, and sometimes even inside the human body. Implantable biosensors are an important class of biosensors based on their ability to continuously measure metabolite levels, without the need for patient intervention and regardless of the patient’s physiological state (sleep, rest, *etc*.) [64]. For example, implantable biosensors represent a highly desirable proposition for diabetes management which currently relies on data obtained by using test strips blood from finger pricking, a procedure which is not only painful, but also is incapable of reflecting the overall direction, trends, and patterns associated with daily habits [65].

This initiated a broad research effort aimed at developing implantable biosensors for continuous monitoring of multiple biologically relevant metabolites, but not wearable sensors. Other classes of implantable devices which have been intensively investigated include sensors for nerve stimulation can ease acute pain sensors to the detecting electric signals in brain and sensors to monitor biological analysis in brain with implanted drug delivery systems for controlled delivery at the site of pain and stress [66].

During the end of the last decade, there has been a significant increase in the number of various wearable health monitoring devices, ranging from simple pulse monitors, activity monitors, and portable Holter monitors, to sophisticated and expensive implantable sensors. The range of implantable biomedical devices will increase substantially over the next decade, thanks to improved technology in MicroSytems technology achieved during the last decade. IWBANs are more desirable than WWBAN for many advantages. WWBANs have some disadvantages: they limit the mobility of the patients; in addition, they can cause skin infections, thus contributing poor health conditions. Despite the wireless connection is not an important requirement for monitoring physiological parameters from implanted sensors. This problem is considered as one of the main motivations for the trend in modern biomedical implanted systems to use wireless technology [66].

Tang *et al*. [67] explored a vision of the near future when one single device will be able to create a WSN with a large number of nodes, which are hosted within and outside the body may be either predetermined or randomly, in accordance with the application. This vision can only be achieved through a broad communication standard for wireless telemetry link. Standard hardware and software architecture can support compatible devices, which are expected to significantly affect next generation of healthcare systems. Some of these devices can then be integrated into the wireless body area network, providing new opportunities for technology to monitor the health status [68].

**Figure 7.**
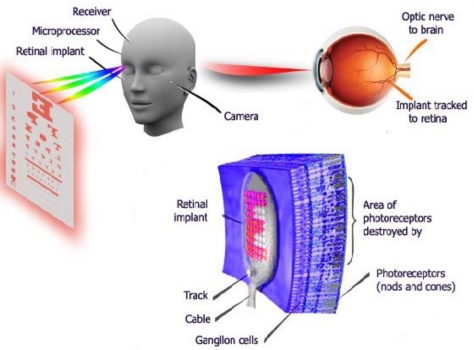
Implantable artificial retina for blind people.

There are a large number of researches which deal with implantable body sensor networks such as [69,70]. One of the prominent applications of implantable sensor networks in healthcare is that IWBAN can help blind people [19] to improve their vision. Patients with no vision or visually impaired can see at a reasonable level by using retina prosthesis chips implanted within a human eye, as shown in Figure 7.

### 4.4. New Sensors Generations for BASNs

In bio-monitoring, the “physical world” which should be monitored is a living body. Thus, the sensor nodes should be placed in close proximity of the body of topic or implanted into the body, and they constitute a body-area network. The evolution of sensor nodes for bio-monitoring is driven by the “disappearance” requirement, and it leverages all technology options available, from the ever-shrinking standard microelectronic technology, to the emerging microfabrication processes allowing the integration of heterogeneous devices onto a small physical volume. As a result, we can clearly see a direction of rapid evolution as in [71]. In the next we will overview four new sensor generations for BASNs.

#### 4.4.1. Obtrusive Devices

These devices are constantly perceived the target topic, because of their size and weight is great enough to be a source of discomfort. Nevertheless, they are portable and they can communicate with other nodes or data collection gateways. The current commercial sensors are obtrusive: examples include the cause electro-cardiographs and tracking systems of the body on the basis of a wearable cameras and a marker. Obtrusiveness is dictated mainly because of two key issues: the high power dissipation, which requires a large battery and/or a short period of time between recharges and cumbersome sensory interface.

#### 4.4.2. Parasitic Devices

These nodes are perceived by the subject as physical objects, but their size, weight and structure do not pose serious impediments to normal behavior. Examples of parasitic devices are bio-metric watches and body-tracking inertial sensors. The physical volume of these nodes must not exceed a few cubic centimeters, and their weight must be in the order of tens of grams. Considering the volumetric energy density of current battery technology, the power consumption of these nodes must not be larger than a few milliwatts. Various parasitic nodes have been recently commercialized, and these devices represent the current state-of-the-art in WSNs.

#### 4.4.3. Symbiotic Nodes

The transition from the state of the art, the scientific community is pushing to more aggressively scaled cubic millimeter size devices (called “smart dust” [72]), which may support a number of new in-body bio-monitoring applications. The technical problems to be addressed are, first, the implementation of self-powered nodes that can collect energy from the body (temperature gradients, movement, in-body chemical reactions, *etc*.). Second, the size limitation imposes demanding requirements on the process of integration and microfabrication: wireless communications, electronic data processing, chemical data processing, microfluidic capabilities should all be packed in a few cubic millimeters. Finally, further problems arise in connection with the safety requirements: nodes must be short and long term bio-compatible. We call these nodes symbiotic because they have a true mutual advantage relationship of the target organism.

#### 4.4.4. Bio-Inspired Nodes

As the end point of the evolution trends, we envision bio-inspired units (nodes), both from the architecture and technology viewpoint. The physical scale of these devices approaches a few cubic microns (or less), and the interaction between the target sensor and the sensor itself disappears. Molecular engineering and nanotechnology will make these devices a reality in the near future. Some research efforts showed that some of the features required in the sensor node can be realized by molecular-scale devices, which are often engineered with the use of bio-molecules. These devices operate autonomously, powered by chemical reactions inspired by biological systems. The process of construction and architecture of these devices will also resemble natural processes in biology: bottom-up self-assembly, self-reproduction and self-repair will be necessary in addition to the safety and biocompatibility.

## 5. Challenges, Open Research Problems, and Future of WBANs

In this section we present the challenges observed in the design pervasive healthcare systems and introduce open problems in research of the wearable and implantable sensor systems. There are many challenges of WSNs at all levels. In the following subsections we will approach these problems from medical [73] and low perspectives, mainly IWBANs. As well, we introduce the future work of WBANs. The stated challenges are selected to be studied to fully exploit the widespread benefits of pervasive healthcare systems through WSNs.

### 5.1. Challenges and Open Research Problems of WBANs

Recent technological advances in sensors and low-power integrated circuits and wireless communications have allowed the design of low cost, miniature, lightweight, and intelligent physiological sensor nodes. These nodes are capable of sensing, processing and communication of one or more vital signs can be easily integrated into wireless personal area networks or the authority to monitor the health condition. These networks promise to revolutionize health care by allowing non-expensive, non-invasive, continuous, ambulatory health monitoring with almost real-time updates of medical records via the Internet. Although the number of research efforts is focusing on various technical, economic, and social issues, many technical hurdles still remain to decide whether to have a flexible, reliable, secure, and energy efficient WBAN suited of medical applications. In this subsection, an overview of hardware and software platforms for the problem of medical monitoring using WBANs is presented. As well as, we will introduce some open problems, and introduce the authors’ suggested solutions for these problems.

#### 5.1.1. Physical Challenges

##### a. Unobtrusiveness

The design and development of wearable sensor devices without violating their unobtrusiveness remains a challenge. When patients have to carry sensors attached on their bodies as in the fall detection system described in [74] and FireLine [75], unobtrusiveness becomes a serious problem among many others challenges. The need for integrating various sensors into a single solution makes both the sensor units of LiveNet [76] and paths. These body-worn sensor devices are heavy and very intrusive devices, while the bandage type electrocardiograms sensors described in [77] and watch-shaped activity recorder in [78] are much easier to bear devices.

##### b. Sensitivity of Sensors

The sensitivity of the sensor devices is especially important when users wear these sensors in harsh environments such as in fire situations. Sweat can affect the transducers of the sensor devices negatively, causing the reduction in the sensitivity of the body-worn sensors or requiring recalibration of the sensors. Gietzelt *et al*. [79] proposed an automatic self-calibration algorithm for triaxial accelerometers. Yet, the self-calibration and sensitivity enhancement algorithms are still needed for sensor devices different than accelerometers. Low-maintenance and highly sensitive vital signs monitoring sensors will attain importance as pervasive healthcare systems evolve.

##### c. Energy of Batteries

Energy in WBANs is considered as one of the crucial problems and challenges and deeply needs investigations and solutions. For indoor areas, like in hospitals, the batteries can be a remedy in some cases, while recharging the batteries can be burdensome, especially for older people. Given the likelihood of forgetting to recharge the batteries of multiple sensors, is an important issue to be resolved. Although there is a lot of an effort design low-power sensors to solve this problem [77], we still need robust energy techniques which is related to the new term “green technology”. The solar cells that can provide up to 15 m under direct sun cannot be used with body-worn wireless sensors because sensors are preferably to be placed under the clothing. Thus, the motion [80] and body heat [81,82] based energy techniques should be explored for healthcare systems.

##### d. Effective Methods for Data Collection

The rate of data collection in pervasive BSANs and healthcare systems is high. Developing effective methods of data processing techniques are considered to be important issue. In some cases 3-lead electrocardiograms cannot be sufficient to detect heart diseases or a single 3-axes accelerometer cannot classify all activities of people. In these cases, more sensors will be required, and the data collection needs will increase. The real time of data collection and analysis of physiological data is essential. In addition, time-stamping and ordering of events, synchronization of different sensors are open problems for study and research. Finally, another important related issue is the integration of various types of sensors, like RFID tags, implantable sensors and wireless body sensors that necessitates the development of modular architectures for further development.

##### e. Reliable Transfer of Data

The low transmission power and small sized antennae of wireless sensor devices can cause reduced signal to noise ratios that cause a higher bit error rate and reduce the reliable coverage area. However, reliable data transfer of data in WBANs and medical monitoring systems is crucial. Thus, the error of network coding schemes is resilient for medical data transfer must be developed to improve network reliability. Marinkovic *et al*. [83] proposed a network coding technique for a TDMA protocol. This allows each sensor to transmit data via two relays and relay nodes XOR packets before sending. Although they showed improvement in the rate of packet by simulating real deployments to measure physiological signals such as electrocardiograms and electroencephalography and can improve the proposed system, respectively, remain as future work. The reliable data transmission should be investigated for low power body sensor networks which still a challenge for BASNs.

##### f. Compatibility Problems

The integration of multiple sensory devices that operate at different frequencies increases the compatibility problems. Communication between devices takes multiple bands and use different protocols. This can lead to interference between different devices, especially in the unlicensed industrial, scientific and medical band radio. WBANs and the widespread healthcare systems must be developed to ensure compatibility between different devices.

##### g. Bandwidth Problems

The bandwidth available for data transmission for WBANs is usually low. Although, the new sensor nodes can operate at 250 Kbps, due to duty cycling mechanisms for lowering the power consumption lowers the actual available bandwidth. If the sensor nodes operate with a duty cycle of 10%, then they are actually transmitting data only 10% of the time. This situation may pose difficulties especially when there is huge amount of data like in the case of transmission of medical diagnostic imaging data, which may require up to Mbit/s level capacities. For this reason the efficient compression algorithms must be developed for multimedia data transmission [84–88]. These compression methods should also be lightweight enough for running on a sensor node that has no high performance processing and memory capabilities.

#### 5.1.2. MAC Layer Challenges

In addition to the usual problems of WSN MAC layer such as energy efficiency [84], there are still some critical and important issues that are specific to WBANs and health monitoring. To begin with, Quality of Service requirements of emergency traffic should be explored for health monitoring applications. Benhaddou *et al.* [85] proposed a MAC scheme for healthcare applications which merge a preemptive service scheduling algorithm into the 802.11e Quality of Service MAC to provide the highest and preemptive channel access precedence for medical emergency traffic. The Quality of Service-aware MAC protocols [89] for low power wireless sensor networks such as 802.15.4 still need research from a healthcare specific perspective. WBANs and Healthcare monitoring applications require emergency event reporting besides periodic physiological data reporting.

Under emergency conditions, the emergency data should be guaranteed to be delivered with a reasonable delay. For this purpose, emergency data prioritization mechanisms should be developed. Moreover, the fairness among different emergent situations should be considered. The event based fairness scheme proposed by Durmus *et al*. [90] is an example for video sensor networks. The prioritization and fairness mechanisms for vital signs monitoring applications are open research issues.

#### 5.1.3. Network Layer Challenges

Energy-efficient techniques used in WBAN networking have recently acheived considerable importance and are defined as green technology. Reducing the energy consumption of computing and communication infrastructure in WBSNs environments is an area of increasing importance for researchers and presents an open research challenges for applications of wireless sensor networks for WBANs and healthcare monitoring systems. The convergent traffic inherent in wireless sensor networks may cause choke effect at the nodes closer to the base station. For this reason, load balancing routing protocols need to be developed. Moreover, when multimedia traffic is encountered with the emergence of multimodal sensor networks for healthcare monitoring applications, congestion avoidance and rate control issues become significant [91]. These techniques should also be integrated with data compression techniques for better utilization.

There is also a problem of sensor nodes’ temperature *in vivo* sensor networks, as they perhaps harm the tissue when they are overheated. To overcome this problem, Tang *et al*. [92] proposed a thermal-aware routing protocol for implantable biosensor networks. These protocols should avoid the degradation of delay performance. However, for reliable data delivery, multipath routing protocols for medical applications based on sensor networks should be studied.

#### 5.1.4. Transport Layer Challenges

WBANs deals with life-critical data, therefore a lost frame or packet of data can cause an alarm situation to be missed totally or misinterpreted. Therefore, reliable data delivery is needed. Although there are reliability procedures at different layers such as automatic repeat request at MAC layer, critical WBANs require total end-to-end reliability procedures [93]. The reliability for WBANs may need either packet level or event level solutions. For periodic traffic, packet level reliability is essential whereas for emergency event reporting such as a sudden fall detection then event reporting is more important than individual packet reporting. Consequently, we need to design a cross layer protocols for ensuring reliable delivery for different types of traffic is also important.

The congestion and flow control procedures at the transport layer are also rare. For instance, the Event-to-Sink Reliable Transport protocol aims to guarantee delivery of enough numbers of packets of data. The Event-to-Sink Reliable Transport protocol is applicable for event reporting applications. On the other hand, for periodic physiological data reporting like electrocardiograms, or heart rate, every single packet has to be delivered reliably; therefore transport protocols addressing this issue still needed to be discussed and developed.

#### 5.1.5. Application Layer Challenges

We have already mentioned and analyzed some of the challenging problems in the previous subsections. One of the main important challenges at the application layer for WBANs is how to produce meaningful information that can be translated into knowledge. As we know, the application layer being at the top surface is expected to have a coordinating mission too. In this context, the organization of ambient sensor data, medical data and other should be held by the application layer. The organization of data is critical and must be explained in more details. Therefore, researchers need to design efficient machine learning algorithms for self-learning, autonomous systems replacing rule based and static systems to overcome the data organization problem.

#### 5.1.6. Independent Challenges

Independent challenges mean the problems that are not in relation to a specific layer. These challenges and some suggestions for their solutions are provided in this section.

##### a. Security

The security of the sensors, the data collection, and the communication of that data to a collection point is an important and critical issue in WBANs, especially for sensitive applications. Some solutions have been proposed to protect the node-to-node communication from any attacks in WBANs. As in any secure system, fundamental security specifications are needed for WBANs which are confidentiality, integrity, availability, accountability, and access control. For assuring these security requirements, new encryption methods should be developed. For instance, in [94] elliptic curve cryptography for key distribution to decrease energy consumption is proposed. However, there is still a need for more efficient cryptography methods to meet the security requirements. Another challenging issue with the security requirements for WBANs arises when the patients are unconscious as in that case obtaining passwords may not be possible. In this case, biometric methods can be used for accountability as in [95]. As well as, the physiological signal based authentication method is proposed in [96]. According to the findings of [96], the proposed scheme generates identity information for mutual authentication from an electrocardiogram-like measure at two different parts of the body. Finding unique biometric features can be used for identification purposes which represent a new challenge in this issue. As well as, when sensors will become smaller and more capable, their benefits will increase. Therefore, the security of the networks they form is critical to maintain, and this will continue to be an important area of research.

##### b. Privacy

Privacy protection of patients in WBANs has been extensively researched and developed. Authorization of the users in the WBANs system should not be overlooked and the users should have autonomy and control over their data of any type [97]. In addition to, especially in image processing applications which are becoming more available from day to day, the privacy-preserving methods should be developed for the comfort of the monitored people. On-node processing of images can be a solution in which no images are transferred, only the information about the image is sent over the network. Srinivasan *et al*. [98] has discussed a wireless sensor system privacy leak called Fingerprint and Timing-based Snooping attacks that can happen even if the wireless communication is encrypted.

##### c. User-Friendliness

WBANs users should include systems for complete satisfaction. Casas *et al*. [99] proposed a user modeling scheme for improving user interaction. The development of natural interfaces between different groups of people and pervasive systems is crucial. In [100], the authors surveyed their level of user-friendliness among a small elderly group and concluded that their interface should be revisited. The needs for different groups should be identified clearly, for instance, patients with cognitive disabilities have different interaction characteristics with the system than patients with diabetes. Moreover, the user interface for the handicapped and the elderly must be based on voice, gesture, and visual animation, and must avoid any kind of particular skills. The interface for healthcare professionals should output medical data such as emergency situation indicators and behavioral patterns of the people under observation in a domain-specific notation. The healthcare professionals and caregivers should also have user-friendly and natural interfaces with immediate response capabilities.

##### d. Ease of Deployment and Scalability

Similar to user-friendliness, developing easily deployable pervasive systems is also an important and nontrivial problem for WBANs. When the number of patients and caregivers increase, the scalable and easily deployable applications that can also support multiple receivers will attain much importance.

WBANs and pervasive health monitoring systems typically require the simultaneous use of several sensors, communication devices and software. With these diverse components, ease of deployment becomes a problem to be considered for research.

For this purpose, a software as a service approach can be used for both scalability and ease of deployment, together with small and easily configurable sensors. The system must support the addition of new components at runtime, in order to adapt the system to changing disabilities over time. The software platforms and distributed services will be required for seamless integration of the hardware and the application levels and interoperability among these will be essential as in [79,101].

##### e. Mobility

The purpose of WBANs and health monitoring systems is to let people go about their life while accessing high-quality medical services. The use of sensor networks for this purpose is not new [102,103]. However, the emergence of WBANs has enabled the development of applications to ensure and promote the mobility of users. Thus, the WBANs technologies enable ubiquitous of healthcare systems. Mobility requires the development of multi-hop, multi-modal, *ad hoc* sensor networks which bring the problems mentioned in the previous subsections along with the problem of location awareness.

#### 5.1.7. Bio-Inspired Sensors

Some of the most exciting and challenging areas of applications of the future, WBANs are deeply related to areas like bioscience (life sciences), biotechnology and nanoscience (nanotechnology). We can mention for example: (i) point-of-care portable or in-body, easy-to-use, stand-alone systems to perform medical analysis out of clinical laboratories [104]; (ii) *in vivo* controlled drug release systems, which can be ingested into a human body and which must act to deliver appropriate quantity of drugs or other caring means in a pre-determined, self-regulating or real time-controllable way [105,106].

In order to be adapted to these applications, the system should be autonomous in terms of avoiding as much as possible direct contribution to the human operator to their functions, which can be achieved by integrating various devices and circuits for processing data and in terms of power supply. In addition, a key factor for the development will be able to connect them to obtain information and manage them from remote.

Hardware component for medical and biological applications has to consider bio-compatibility problems and the high specific properties of the interaction at the molecular and atomic level. This can be achieved through development of technologies, such as: (a) advanced synthetic or non-molecular receptors [107]; (b) innovative three-dimensional membranes for highly-molecular controlled release [108]; (c) bio-hybrid systems as bio-coated nanoparticles for a vehicle of drugs or transfection agents inside the cell [109]; (d) bio-inspired systems to simulate specific features of biological systems as adaptation and self-regulation [110]; (e) smart surfaces for organic transduction electronically specific mechanical or optical stimuli [111].

It is clear that the high bio-nanoscience content involved in the design of new WBANs systems will fuel innovation in many fields as molecular sensing, new transduction techniques of physical signals, new smart, multi-functional materials.

The conceivable evolution of sensor systems for WBANs could be depicted as developed along two phases. The first one is the application of conventional (micro) and non-conventional (nano) technology to the bio-physical world and, in particular, by patterning and disposing biological matter and by handling fluids in a microscopic, high-controlled way to separate and process small volumes of sample or selectively interact with single cells. The second phase will be dominated by the exploitation of bioscience for the creation of micro and nanoscale systems and materials with unique properties and functions. These systems will be characterized by properties of self-assembly, reversibility, adaptability, self-replication, possibility of interaction at the atomic scale [69,112].

### 5.2. The Future of WBAN Systems

In order to exploit the features and benefits of WBANs in the future for the medical environment, it must include the following significant improvements design and features according to [113–116]:

- Low cost and low size sensor node electronics design with wireless capability. Sensor nodes will be able to transmit data at a distance of several meters. Sensor nodes must be miniaturized so that they can be easily wearable or implantable.
- Energy optimization methods should be developed by the combination of the link and physical layer functionality for wireless devices, which leads to provide longer battery lifetime and thus extends the lifetime of the sensor nodes. Sleep mode method should be used so that the sensor nodes will spend most of their time in a state of low consumption of electricity, when data transmission is not required.
- Physiological data must be classified as crucial and noncrucial data for each patient. As an example, although it may be different with different patients, vital signals as electrocardiograms may be more important than the temperature for some patients. Thus WBANs system must give priority to the important data.
- High gain miniature antennas should be designed for sensor nodes to increase the transmission reliability and to minimize interference, thus reducing the energy consumption.
- Each sensor must be optimized in accordance with its characteristics using a variable sampling rate.
- Unlike other systems, in the sensor network in a WBAN systems, each sensor signal has a different frequency (*i.e*., not uniform) and thus each sensor node must be optimized in accordance with to the frequency band of its sensor. In addition, given that some not important physiological signals such as temperature information can only be measured over longer period of time, WBANs will lead to better performance, if the “adaptive” communication protocol is used to accommodate such differences in the system, which will certainly make the system efficient power.
- In order to ensure continued remote monitoring of WBANs, multiple gateway devices must be designed to interface with existing wireless systems in healthcare area. These gateways will mainly be used for communication between the CCUs and the remote computers or mobile devices.
- A transmission mechanism should be integrated into WBANs, which could be useful for the free movement of patients in large medical applications. It can be used to track patient throughout a hospital or the patient can keep track of the places where they are outdoors doing their daily activities. An alarm feature can be enabled if the patient leaves the room or goes out of range of a CCU. This may give the last location of patients, which would allow staff to easily track the location of the patient location. Such features could be incorporated into the detection system of signals through multiple remote stations, which are generally known as soft handover.
- Additional essential component in WBANs is security. Basic software components must be identified and developed to accommodate secure and efficient wireless network. Data should be only accessible by only an authorized person in remote destinations. Hardware components and software programs should be coordinated together to provide secure and reliable communications.
- Personal health monitoring systems were used only for data collecting. Data processing and analysis are performed offline, which makes such devices impractical for continuous monitoring and early detection of health disorders.
- Systems with multiple sensors for physical rehabilitation often feature unwieldy wires between the sensors and the monitoring system. These wires may limit the activity of the patient and level of comfort and thus negatively affects the measurement results.
- Individual sensors often operate as standalone systems in WBANs and usually do not offer the flexibility and integration with third-party devices.
- Eventually, the WBANs systems should have their own standards for data collection and storing methods, as well as for wireless communication to eliminate coexistence issues.

## 6. Conclusions and Ongoing Work

This paper shows that WBANs can be widely used in medical applications. It has surveyed a number of systems and applications for healthcare, mainly wearable and implantable sensors, and describes the major challenges and open research problems of WBANs. As nanotechnology provides smaller and multiple-functional sensor nodes, the further these new small body networks may evolve, making their use as natural as wearing cloth in the near future.

Given the importance of addressing ways to provide smart healthcare for the elderly, chronically ill and children, researchers have started to explore technological solutions to enhance the provision of health and social care in a way which complements existing services. In this paper, we have presented some applications of how people could benefit from living in homes that have wireless sensor technologies for improving the quality of life and outlined issues to keep in mind during their development.

The opportunities are immense. WSNs can open a huge market of consumer applications, mainly in healthcare monitoring and environmental applications. In the near future, the evolution of WBANs for symbiotic and bio-inspired architectures can significantly improve the health conditions and lifetime expectation for a large number of people. Remote monitoring of patients, powered by the advent of mobility systems, is not a new idea. However, wireless sensor networks provide a low cost means to sense a given environment and thanks to its wireless nature, it proves to be adequate for unobtrusive deployment on the patient. In the near future, in healthcare there will be multi-modal sensor solutions which include the benefits described in this paper; however, there are still challenges to overcome to achieve these context-aware, pervasive healthcare applications. We have provided an analysis of these challenges from a healthcare perspective of WSNs. A combination of different forms of sound modalities like video sensing and medical sensors along with smart devices and remote monitoring capabilities will lead context-aware, pervasive healthcare applications to become within the reach of ordinary users.

Finally, we generalize assembled a list of current sensor networks research problems, which provide more opportunities and advantages of the WBANs systems. Along with the current problems of research, the authors encourage a deeper understanding of the problems and a lot of more development in solutions to the open research problems, as we have described in this paper.

- Transport, network, data link and physical layers, and application layers
- Power control, mobility and task management planes
- Distributed network and Internet access to sensors, controls, and processors
- Data dissemination protocols
- Security protocol
- Information networking architecture
- Framework for implementing adaptive energy-aware distributed microsensors
- Cluster formation protocol
- Laser communication from a cubic millimeter
- Scalable coordination architectures for deeply distributed and dynamic systems
- Routing and power aware sensor management
- Distributed query processing
- Mathematical framework that incorporates key features of computing nodes and networking elements
- Computation is directly limited due to the limited amount of power. Typically, biosensors are not expected to have the same computational power as conventional WSN nodes.
- Material constraints are another issue for wireless sensor networks application to WBANs and healthcare applications. A biosensor must be in contact with human body, or even on it. If the biosensor is inside a pill, the choice of construction materials must be careful, especially on batteries. Also chemical reactions with body tissue and the disposal of the sensor are of utmost importance.

The authors believe that the role of WBANs in medicine can be further enlarged. In the near future, the use of WBANs will increase because smart spaces will be enabled with wireless sensor networks which can sense environmental conditions and take preventive actions based on the presence of humans is those spaces. The system can therefore reach ubiquity, where each individual would have a computational module able to seamlessly interact with the smart space’s system and prevent health problems.
