# Correction: Darwish, A. and Hassanien, A.E. Wearable and Implantable Wireless Sensor Network Solutions for Healthcare Monitoring. 

**DOI:** 10.3390/s120912375

**Published:** 2012-09-12

**Authors:** Ashraf Darwish, Aboul Ella Hassanien

**Affiliations:** 1 Faculty of Science, Helwan University, Cairo, Egypt; 2 Faculty of Computers and Information, Cairo University, Cairo, Egypt; E-Mail: aboitcairo@gmail.com

A reference is missing in our paper [1]. Figure 2 was adapted from Reference [2] with permission. The figure is listed and described as below:

WBANs application in the medical field are composed of wearable and implantable sensors that can detect information from the human body and send it to a central unit as shown in Figure 2. These sensors have some characteristics such as small, low-power detection and have the capability to detect medical signals data from the control unit. There is a difficulty in the monitoring devices that are not completely wearable where the wires are used to connect many sensors. Yuce [2] explored a vision to the future of medical sensor networks should be miniaturized and also wearable sensors that can communicate with the receiving device wirelessly.

## Figures and Tables

**Figure 2.**
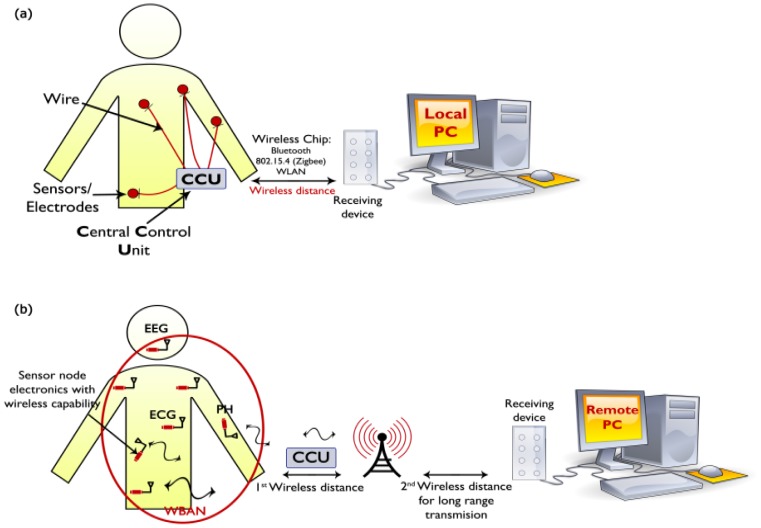
A WSN system: (**a**) wired system and (**b**) wireless system. Adapted with permission from [2].
